# Measuring Coverage in MNCH: Population HIV-Free Survival among Children under Two Years of Age in Four African Countries

**DOI:** 10.1371/journal.pmed.1001424

**Published:** 2013-05-07

**Authors:** Jeffrey S. A. Stringer, Kathryn Stinson, Pius M. Tih, Mark J. Giganti, Didier K. Ekouevi, Tracy L. Creek, Thomas K. Welty, Benjamin H. Chi, Catherine M. Wilfert, Nathan Shaffer, Elizabeth M. Stringer, Francois Dabis, David Coetzee

**Affiliations:** 1 Centre for Infectious Disease Research in Zambia, Lusaka, Zambia; 2 Department of Obstetrics and Gynecology, University of North Carolina School of Medicine, Chapel Hill, North Carolina, United States of America; 3 Centre for Infectious Disease Epidemiology and Research, School of Public Health and Family Medicine, University of Cape Town, Cape Town, South Africa; 4 Cameroon Baptist Health Convention Health Board, Bamenda, Cameroon; 5 Programme PAC-CI, Abidjan, Côte D'Ivoire, France; 6 Institute National de la Santé et de la Recherche Médicale, Université Victor Segalen, Bordeaux, France; 7 Children's Healthcare of Atlanta, Atlanta, Georgia, United States of America; 8 Elizabeth Glaser Pediatric AIDS Foundation, Washington, District of Columbia, United States of America; 9 World Health Organization, Geneva, Switzerland; Independent consultant: Health statistics and policy, Switzerland

## Abstract

**Background:**

Population-based evaluations of programs for prevention of mother-to-child HIV transmission (PMTCT) are scarce. We measured PMTCT service coverage, regimen use, and HIV-free survival among children ≤24 mo of age in Cameroon, Côte D'Ivoire, South Africa, and Zambia.

**Methods and Findings:**

We randomly sampled households in 26 communities and offered participation if a child had been born to a woman living there during the prior 24 mo. We tested consenting mothers with rapid HIV antibody tests and tested the children of seropositive mothers with HIV DNA PCR or rapid antibody tests. Our primary outcome was 24-mo HIV-free survival, estimated with survival analysis. In an individual-level analysis, we evaluated the effectiveness of various PMTCT regimens. In a community-level analysis, we evaluated the relationship between HIV-free survival and community PMTCT coverage (the proportion of HIV-exposed infants in each community that received any PMTCT intervention during gestation or breastfeeding). We also compared our community coverage results to those of a contemporaneous study conducted in the facilities serving each sampled community. Of 7,985 surveyed children under 2 y of age, 1,014 (12.7%) were HIV-exposed. Of these, 110 (10.9%) were HIV-infected, 851 (83.9%) were HIV-uninfected, and 53 (5.2%) were dead. HIV-free survival at 24 mo of age among all HIV-exposed children was 79.7% (95% CI: 76.4, 82.6) overall, with the following country-level estimates: Cameroon (72.6%; 95% CI: 62.3, 80.5), South Africa (77.7%; 95% CI: 72.5, 82.1), Zambia (83.1%; 95% CI: 78.4, 86.8), and Côte D'Ivoire (84.4%; 95% CI: 70.0, 92.2). In adjusted analyses, the risk of death or HIV infection was non-significantly lower in children whose mothers received a more complex regimen of either two or three antiretroviral drugs compared to those receiving no prophylaxis (adjusted hazard ratio: 0.60; 95% CI: 0.34, 1.06). Risk of death was not different for children whose mothers received a more complex regimen compared to those given single-dose nevirapine (adjusted hazard ratio: 0.88; 95% CI: 0.45, 1.72). Community PMTCT coverage was highest in Cameroon, where 75 of 114 HIV-exposed infants met criteria for coverage (66%; 95% CI: 56, 74), followed by Zambia (219 of 444, 49%; 95% CI: 45, 54), then South Africa (152 of 365, 42%; 95% CI: 37, 47), and then Côte D'Ivoire (3 of 53, 5.7%; 95% CI: 1.2, 16). In a cluster-level analysis, community PMTCT coverage was highly correlated with facility PMTCT coverage (Pearson's *r* = 0.85), and moderately correlated with 24-mo HIV-free survival (Pearson's *r* = 0.29). In 14 of 16 instances where both the facility and community samples were large enough for comparison, the facility-based coverage measure exceeded that observed in the community.

**Conclusions:**

HIV-free survival can be estimated with community surveys and should be incorporated into ongoing country monitoring. Facility-based coverage measures correlate with those derived from community sampling, but may overestimate population coverage. The more complex regimens recommended by the World Health Organization seem to have measurable public health benefit at the population level, but power was limited and additional field validation is needed.

*Please see later in the article for the Editors' Summary*

## Editors' Summary

**Background:**

For a pregnant woman who is HIV-positive, the discrepancy across the world in outlook for mother and child is stark. Mother-to-child transmission of HIV during pregnancy is now less than 1% in many high-income settings, but occurs much more often in low-income countries. Three interventions have a major impact on transmission of HIV to the baby: antiretroviral drugs, mode of delivery, and type of infant feeding. The latter two are complex, as the interventions commonly used in high-income countries (cesarean section if the maternal viral load is high; exclusive formula feeding) have their own risks in low-income settings. Minimizing the risks of transmitting HIV through effective drug regimes therefore becomes particularly important. Monitoring progress on reducing the incidence of mother-to-child HIV transmission is essential, but not always easy to achieve.

**Why Was This Study Done?:**

A research group led by Stringer and colleagues recently reported a study from four countries in Africa: Cameroon, Côte D'Ivoire, South Africa, and Zambia. The study showed that even in the health facility setting (e.g., hospitals and clinics), only half of infants whose mothers were HIV-positive received the minimum recommended drug treatment (one dose of nevirapine during labor) to prevent HIV transmission. Across the population of these countries, it is possible that fewer receive antiretroviral drugs, as the study did not include women who did not access health facilities. Therefore, the next stage of the study by this research group, reported here, involved going into the communities around these health facilities to find out how many infants under two years old had been exposed to HIV, whether they had received drugs to prevent transmission, and what proportion were alive and not infected with HIV at two years old.

**What Did the Researchers Do and Find?:**

The researchers tested all consenting women who had delivered a baby in the last two years in the surrounding communities. If the mother was found to be HIV-positive, then the infant was also tested for HIV. The researchers then calculated how many of the infants would be alive at two years and free of HIV infection.

Most mothers (78%) agreed to testing for themselves and their infants. There were 7,985 children under two years of age in this study, of whom 13% had been born to an HIV-positive mother. Less than half (46%) of the HIV-positive mothers had received any drugs to prevent HIV transmission. Of the children with HIV-positive mothers, 11% were HIV-infected, 84% were not infected with HIV, and 5% had died. Overall, the researchers estimated that around 80% of these children would be alive at two years without HIV infection. This proportion differed non-significantly between the four countries (ranging from 73% to 84%). The researchers found higher rates of infant survival than they had expected and knew that they might have missed some infant deaths (e.g., if households with infant deaths were less likely to take part in the study).

The researchers found that their estimates of the proportion of HIV-positive mothers who received drugs to prevent transmission were fairly similar between their previous study, looking at health facilities, and this study of the surrounding communities. However, in 14 out of 16 comparisons, the estimate from the community was lower than that from the facility.

**What Do These Findings Mean?:**

This study shows that it would be possible to estimate how many infants are surviving free of HIV infection using a study based in the community, and that these estimates may be more accurate than those for studies based in health facilities. There are still a large proportion of HIV-positive mothers who are not receiving drugs to prevent transmission to the baby. The authors suggest that using two or three drugs to prevent HIV may help to reduce transmission.

There are already community surveys conducted in many low-income countries, but they have not included routine infant testing for HIV. It is now essential that organizations providing drugs, money, and infrastructure in this field consider more accurate means of monitoring incidence of HIV transmission from mother to infant, particularly at the community level.

**Additional Information:**

Please access these websites via the online version of this summary at http://dx.doi.org/10.1371/journal.pmed.1001424.

- The World Health Organization has more information on mother-to-child transmission of HIV
- The United Nations Children's Fund has more information on the status of national PMTCT responses in the most affected countries

*This paper is part of the* PLOS Medicine “*Measuring Coverage in MNCH” Collection.*

## Introduction

While transmission of HIV from mother to infant has been virtually eliminated in high-income countries, substantial challenges remain in low- and middle-income settings, where almost 1,000 children are thought to acquire HIV each day [1]–[3]. The efficacy of antiretroviral (ARV) regimens during pregnancy and postpartum to prevent mother-to-child transmission is well established, and prevention of mother-to-child transmission (PMTCT) programs have been implemented in most countries in Africa [4],[5]. However, objective measures of program effectiveness and public health impact are largely absent from the refereed literature [6],[7]. Despite calls for the virtual elimination of pediatric HIV, global coverage of PMTCT services remains far below what will be required to meet such an aggressive goal [8]. Current ARV guidelines for resource-limited settings recommend earlier and more effective regimens at higher CD4+ T-lymphocyte thresholds in pregnancy, as well as postpartum treatment or prophylaxis to the mother or child to prevent transmission through breastfeeding. More recently, the initiation of lifelong highly active antiretroviral therapy (HAART) has been proposed for all pregnant, HIV-infected women [9]–[11].

The performance of national PMTCT programs is commonly assessed by aggregating routinely collected service coverage indicators, which are often incomplete and inaccurate, and typically lack the site-level nuance needed to drive meaningful quality improvement efforts [12]–[14]. Recent service-based evaluations have used umbilical cord blood surveillance [15],[16] and infant PCR testing at immunization clinics [17],[18] to improve data quality, but since these methods are facility-based they may not represent the entire community [6].

## Methods

The PMTCT Effectiveness in Africa: Research and Linkages to Care Study (PEARL Study) was a multi-country evaluation of the effectiveness of PMTCT services at the community and facility level. The study's methodology and facility-based components have been reported elsewhere [6],[15],[19]–[21]. The community survey component of the study was conducted in 26 communities in Cameroon, Côte D'Ivoire, South Africa, and Zambia between April 2007 and May 2009.

### Household Selection

Each country employed a two-stage sampling technique. The first stage involved random selection of facilities providing PMTCT services and has been described in detail elsewhere [15]. Once the health facilities were selected in the first stage of sampling, the catchment area of each facility was defined. In all countries, this was done at the local level by reviewing available district data and consulting with local staff. In South Africa and Zambia, we used publically available satellite images (http://www.google.com/earth); in Cameroon and Côte D'Ivoire, we relied upon local maps. Once the catchment boundaries were defined, we subdivided each evaluation area by overlaying a grid of 1 km by 1 km squares. We then estimated the approximate number of households in each square. If the number of households exceeded 50, the square was further subdivided into four smaller squares. All squares of at least five households were then ranked in random order using a “rank probability proportional to size” technique and were then visited in sequence, with all households in each successive grid approached until at least 250 households had been surveyed. In cases where no one was home to participate in the interview, we made two additional attempts to return, after which we counted the household as unavailable.

Each country team employed different ways to educate the communities prior to the surveys, but in each case we met with local community leaders to introduce the survey. Drama performances at public markets and targeted radio announcement were also used. Households were eligible to participate if a child was born in the home during the 24 mo prior to the study visit (whether the mother or infant was currently alive or dead). If the child's mother was not available for interview, we made up to two additional attempts to find her. Failing that, or in the case of a deceased mother, a caregiver was interviewed.

### Study Questionnaire

The survey instrument was adapted from the Zambia Demographic and Health Surveys (DHS) [22] and comprised three parts. Team members used an eligibility form to enumerate all living and deceased household members, their sex, and their age. If there was a child born in the household within the 2 y prior to the survey, the household was deemed eligible for participation in the study and informed consent was obtained. The primary survey respondent was the mother of the eligible child. If the mother was not available, the child's primary caregiver was asked to participate. The maternal questionnaire focused on household characteristics; demographic information; HIV knowledge, attitudes, and perceptions; and previous obstetrical history. The infant module covered specific information about the index pregnancy, including access to and utilization of PMTCT, delivery and postnatal care, and infant feeding. Teams of at least two members, including at least one registered nurse, were trained to administer the survey in local languages at each site.

### Specimen Collection and Testing

In all countries we tested all participating HIV-exposed infants under 24 mo of age for HIV infection. Our procedures for household testing and results disclosure varied by country. In Cameroon, mothers and children over 1 y of age were antibody tested in the household, where pre- and post-test counseling was performed, and results were provided during the visit. Infant dried blood spots for HIV PCR testing were not collected from children whose mothers were seronegative or from those whose mothers were seropositive, but who were over 12 mo of age, no longer breastfeeding, and themselves HIV antibody negative. In the other countries, specimens were obtained from all consenting mothers and children, with HIV testing performed off-site. In Zambia, we provided a card with a linked identification number that allowed participants to access their results (and/or those of their child) at the local health facility. In South Africa and Côte D'Ivoire, test results were not provided to participants. This was a requirement of local ethics review boards, since we were unable to do confirmatory testing in the home. Women were encouraged to access routine counseling and testing services if they so desired, and the study team provided information about where free services could be accessed. In Zambia, we also offered infant hemoglobin testing with referral for children found to be anemic.

All countries used the Determine HIV-1 rapid antibody test (Abbott Laboratories) for initial testing. Confirmatory tests differed by country. We tested infant dried blood spots for HIV DNA PCR using Roche Amplicor version 1.5 with manual extraction.

### Informed Consent and Ethical Approvals

In each country setting, informed consent was obtained prior to administering the questionnaire and blood draws. The informed consent information was customized to suit the procedure and research ethics requirements in the respective countries. In cases of participant illiteracy, a non-biased literate witness joined the informed consent procedure to ensure that objective information was given about the study and that the patient understood. Ethical approval was obtained from the institutional review boards at the University of Alabama at Birmingham (Birmingham, Alabama, US) and the US Centers for Disease Control and Prevention (Atlanta, Georgia, US), as well as the local research ethics review bodies in each of the participating countries.

### Analysis

Our primary outcome was HIV-free survival among HIV-exposed children. Our sample size was chosen to deliver a half-confidence interval of 5% around the primary outcome in each country; we assumed a binomial distribution. The actual number of houses approached per community also took into account country-specific estimates of maternal HIV seroprevalence and baseline child survival. Statistical analyses were performed using SAS statistical software version 9.1.4 (SAS Institute) and R software version 2.4.1 (http://www.r-project.org). Background characteristics were described using medians and interquartile ranges (IQRs) for continuous variables and percentages for categorical variables. Children were stratified according to their HIV-exposure status, HIV-status, and mortality. The present analysis is limited to those children who were known to be exposed to HIV, either through the detection of HIV antibodies in the infant him- or herself, or through a positive maternal HIV diagnosis. HIV-exposed children who were alive and tested HIV negative by DNA PCR were right-censored at the time of testing in time-to-event analyses. HIV-infected children were left-censored at the time of testing, because transmission was known to have occurred prior to the test date. Twenty-four-month HIV-free survival was estimated using a Weibull regression model and a parametric accelerated failure time model with corresponding 95% confidence intervals. Hazard ratios (HRs) for various covariates of interest were fitted in multivariate Weibull regression models and were restricted to children with responses for each of the covariates of interest. Children born in a particular community or a particular country may be similar to each other. We accounted for this potential correlation by including a separate fixed effect variable for both community and country in our multivariable model. In cases where one mother contributed more than one child to the analysis (e.g., twin gestation or short birth interval), we accounted for this correlation by including a “mother” variable as a random effect. To understand the potential for variable co-linearity, we calculated the variance inflation factors of all variables included in the multivariate model. The variance inflation factor for each variable was less than 1.5, which indicates variable multi-co-linearity is not an issue in our model. The effect of infant feeding on HIV-free survival was limited to those children who were at least 6 mo of age.

In a planned secondary analysis, we compared coverage of PMTCT services at the facility level (“facility PMTCT coverage”) to coverage of PMTCT services at the community level (“community PMTCT coverage”). We also compared facility PMTCT coverage to HIV-free survival among HIV-exposed infants. Facility PMTCT coverage was ascertained through an anonymous cord blood surveillance exercise that has been described elsewhere [15]. In the present report, we compare this facility-based coverage measure to the results of our community surveys. Community PMTCT coverage is defined as the proportion of children in our survey born to HIV-positive mothers in whom any ARV drug is reported to have been used during pregnancy. We weighted our estimates according to the number of HIV-exposed infants in a given service cluster and restricted the analysis to clusters with at least 15 exposed infants.

## Results

**Figure 1:**
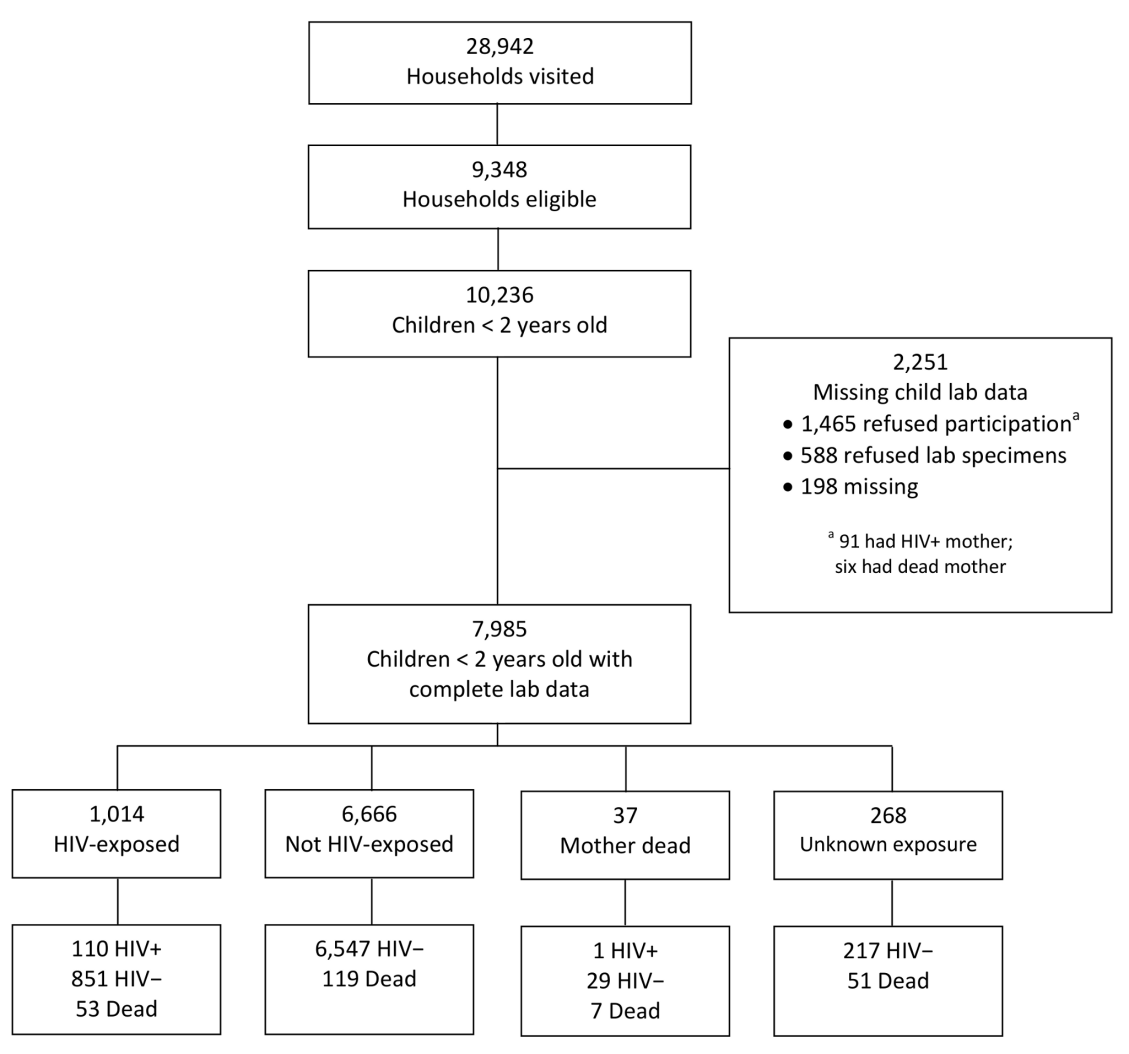
Description of the cohort of children born in the previous 2 y (*n* = 10,236) from all eligible households (*n* = 9,348) visited from May 2008 to May 2009.

The household survey was conducted in six areas in Cameroon, six in Côte D'Ivoire, six in South Africa, and eight in Zambia. Collectively, these areas represent the entire catchment population of the 43 facilities included in the sampling scheme [15]. Of 28,942 households visited between 16 May 2008 and 20 May 2009, 9,348 (32%) were eligible (Figure 1). In total, 10,236 children had been born to 9,606 mothers in the previous 2 y. Some children could not be analyzed: 1,465 (14%) mothers or caregivers refused to participate in the survey, while 588 (6%) agreed to be interviewed but refused to provide specimens. In addition, we lost or could not analyze 198 specimens (2%).

Of 7,985 surveyed and analyzable children under 2 y of age, 1,014 (12.7%) were HIV-exposed. Of these, 110 (10.9%) were HIV-infected, 851 (83.9%) were HIV-uninfected, and 53 (5.2%) were dead (Figure 1). Overall response rates—defined as complete child data available—varied by country, with South Africa having the lowest (64%), followed by Côte D'Ivoire (73%), Zambia (88%), and Cameroon (90%).

### Participant Characteristics

Household characteristics are shown in Table 1. The proportion of households reporting access to a flush toilet or pit latrine ranged from 41% in Côte D'Ivoire to 98% in Cameroon. More than 60% of all households in each of the four countries had finished floors (e.g., cement, tile, or wood). Access to electricity was highest in South Africa and Côte D'Ivoire (95%), and lower in Cameroon (66%) and Zambia (33%). South Africa had the highest proportion of households with a refrigerator (75%) and television (81%). The proportion of homes with mobile phones ranged from 55% in Zambia to 87% in South Africa.

**Table 1:** Characteristics of households surveyed with a child under 2 y of age in the PEARL Study.

Characteristic	Côte D'Ivoire		Cameroon		Zambia		South Africa		Total	
	*N*	Value	*N*	Value	*N*	Value	*N*	Value	*N*	Value
**Participation in survey**	1,682	1,579 (93.9%)	2,425	2,403 (99.1%)	2,302	2,089 (90.7%)	2,939	1,924 (65.5%)	9,348	7,995 (85.6%)
**Water supply to household**	1,561		2,385		2,083		1,915		7,944	
Piped water into house		409 (26.2%)		97 (4.1%)		305 (14.6%)		762 (39.8%)		1,573 (19.8%)
Piped water outside but available within plot		749 (48.0%)		302 (12.7%)		279 (13.4%)		937 (48.9%)		2,267 (28.5%)
Public tap		130 (8.3%)		1,472 (61.7%)		488 (23.4%)		177 (9.2%)		2,267 (28.5%)
Other		273 (17.5%)		514 (21.6%)		1,011 (48.5%)		39 (2.0%)		1,837 (23.1%)
**Toilet facilities in household**	1,556		2,394		2,086		1,921		7,957	
Flush toilet or pit latrine		637 (40.9%)		2,352 (98.2%)		1,844 (88.4%)		1,577 (82.1%)		6,410 (80.6%)
No facility		919 (59.1%)		42 (1.8%)		242 (11.6%)		344 (17.9%)		1,547 (19.4%)
**Does your household have electricity?**	1,566		2,400		2,086		1,924		7,976	
Yes		1,491 (95.2%)		1,597 (66.5%)		685 (32.8%)		1,826 (94.9%)		5,599 (70.2%)
No		75 (4.8%)		803 (33.5%)		1,401 (67.2%)		98 (5.1%)		2,377 (29.8%)
**Does your household have a refrigerator?**	1,563		2,398		2,085		1,923		7,969	
Yes		303 (19.4%)		296 (12.3%)		458 (22.0%)		1,444 (75.1%)		2,501 (31.4%)
No		1,260 (80.6%)		2,102 (87.7%)		1,627 (78.0%)		479 (24.9%)		5,468 (68.6%)
**Main material of floor**	1,557		2,351		2,055		1,917		7,880	
Finished floor (cement/tiles/wood planks)		1,375 (88.3%)		1,593 (67.8%)		1,257 (61.2%)		1,675 (87.4%)		5,900 (74.9%)
Natural floor (earth/mud/dung/sand)		182 (11.7%)		758 (32.2%)		798 (38.8%)		242 (12.6%)		1,980 (25.1%)
**Does your household have a television?**	1,564		2,396		2,059		1,914		7,933	
Yes		1,188 (76.0%)		1,243 (51.9%)		797 (38.7%)		1,558 (81.4%)		4,786 (60.3%)
No		376 (24.0%)		1,153 (48.1%)		1,262 (61.3%)		356 (18.6%)		3,147 (39.7%)
**Does your household have a cell phone?**	1,563		2,393		2,075		1,912		7,943	
Yes		1,205 (77.1%)		1,583 (66.2%)		1,139 (54.9%)		1,664 (87.0%)		5,591 (70.4%)
No		358 (22.9%)		810 (33.8%)		936 (45.1%)		248 (13.0%)		2,352 (29.6%)

The median age of women participating in the survey was 25 y (IQR: 21–30) and the median parity was 2 (IQR: 1–3). With the exception of South African mothers (43%), the majority of women were married or cohabiting. Employment was highest in Cameroon (67%) and lowest in South Africa (20%). In South Africa 83% of mothers had completed at least secondary level school, but only 13% had done so in Cameroon (Table 2).

**Table 2:** Characteristics of mothers surveyed with a child under 2 y of age in the PEARL Study.

Characteristic	Côte D'Ivoire		Cameroon		Zambia		South Africa		Total	
	*N*	Value	*N*	Value	*N*	Value	*N*	Value	*N*	Value
**Participation in survey**	1,869	1,751 (93.7%)	2,451	2,423 (98.9%)	2,347	2,116 (90.2%)	2,939	2,029 (69.0%)	9,606	8,319 (86.6%)
**Caregiver interviewed instead of mother**	1,731	2 (0.1%)	2,412	25 (1.0%)	2,110	18 (0.9%)	2,013	121 (6.0%)	8,266	166 (2.0%)
**Age at time of survey (median [IQR])**	1,730	26 (21, 30)	2,404	25 (22, 30)	2,112	25 (21, 31)	2,026	25 (21, 32)	8,272	25 (21, 30)
12–19 y		227 (13.1%)		276 (11.5%)		285 (13.5%)		312 (15.4%)		1,100 (13.3%)
20–29 y		995 (57.5%)		1,487 (61.9%)		1,211 (57.4%)		1,074 (53.0%)		4,767 (57.6%)
30+ y		508 (29.4%)		639 (26.6%)		615 (29.1%)		640 (31.6%)		2,402 (29.0%)
**Parity (median [IQR])**	1,558	2 (1, 3)	2,394	2 (1, 3)	2,084	2 (1, 4)	1,918	2 (1, 3)	7,954	2 (1, 3)
1		638 (40.9%)		812 (33.9%)		681 (32.7%)		861 (44.9%)		2,992 (37.6%)
2		500 (32.1%)		575 (24.0%)		461 (22.1%)		534 (27.8%)		2,070 (26.0%)
3+		420 (27.0%)		1,007 (42.1%)		942 (45.2%)		523 (27.3%)		2,892 (36.4%)
**Marital status**	1,726		2,414		2,103		2,019		8,262	
Married/cohabitating		1,513 (87.7%)		1,878 (77.8%)		1,655 (78.7%)		870 (43.1%)		5,916 (71.6%)
Other		213 (12.3%)		536 (22.2%)		448 (21.3%)		1,149 (56.9%)		2,346 (28.4%)
**Education**	1,723		2,401		2,100		2,019		8,243	
Secondary or higher		221 (12.8%)		980 (40.8%)		872 (41.5%)		1,682 (83.3%)		3,755 (45.6%)
No schooling or primary		1,502 (87.2%)		1,421 (59.2%)		1,228 (58.5%)		337 (16.7%)		4,488 (54.4%)
**Mother currently employed**	1,562		2,393		2,071		1,921		7,947	
Yes		640 (41.0%)		1,600 (66.9%)		1,136 (54.9%)		372 (19.4%)		3,748 (47.2%)
No		922 (59.0%)		793 (33.1%)		935 (45.1%)		1,549 (80.6%)		4,199 (52.8%)
**Maternal HIV status (lab data)**	1,325		2,247		1,968		1,917		7,457	
Positive		52 (3.9%)		167 (7.4%)		464 (23.6%)		379 (19.8%)		1,062 (14.2%)
Negative		1,265 (95.5%)		2,076 (92.4%)		1,499 (76.2%)		1,515 (79.0%)		6,355 (85.2%)
Mother dead		8 (0.6%)		4 (0.2%)		5 (0.3%)		23 (1.2%)		40 (0.5%)
**PMTCT regimen**	48		160		450		363		1,021	
None		25 (52.1%)		77 (48.1%)		240 (53.3%)		222 (61.2%)		564 (55.2%)
NVP only		1 (2.1%)		37 (23.1%)		48 (10.7%)		62 (17.1%)		148 (14.5%)
NVP+zidovudine		0 (0.0%)		40 (25.0%)		98 (21.8%)		46 (12.7%)		184 (18.0%)
HAART		22 (45.8%)		6 (3.8%)		64 (14.2%)		33 (9.1%)		125 (12.2%)

Data are number (percent) unless otherwise indicated.

Reported utilization of antenatal and delivery services was high across all countries (Table 3). More than 90% of women had attended at least one antenatal visit during their pregnancy with the index child, and the proportion reporting a facility delivery ranged from 87% in Côte D'Ivoire to 96% in Cameroon. The median gestational age at which women initiated antenatal care was 4 mo (IQR: 3–6) in Côte D'Ivoire and South Africa and 5 mo (IQR: 4–6) in Cameroon and Zambia. The proportion of respondents who reported having undergone HIV testing during the index pregnancy was high for Zambia (91%), Cameroon (93%), and South Africa (94%), but much lower in Côte D'Ivoire (39%). Among women who reported having been tested for HIV during the index pregnancy, the rates of self-reported HIV-positivity were significantly lower than the actual HIV seroprevalence of mothers from blood collected at the time of the survey (Côte D'Ivoire [0.3% versus 3.9%; *p*<0.01], Cameroon [4.9% versus 7.4%; *p*<0.01], South Africa [9.1% versus 19.8%; *p*<0.01], and Zambia [12.6% versus 23.6%; *p*<0.01]).

**Table 3:** Characteristics of antenatal and postnatal care for children under 2 y of age in the PEARL Study.

Characteristic	Côte D'Ivoire		Cameroon		Zambia		South Africa		Total	
	*N*	Value	*N*	Value	*N*	Value	*N*	Value	*N*	Value
**Participation in survey among eligible children**	1,934	1,797 (92.9%)	2,601	2,523 (97.0%)	2,441	2,173 (89.0%)	2,145	2,108 (98.3%)	9,121	8,601 (94.3%)
**Gestational age at which ANC was sought (median [IQR])**	1,549	4 (3, 6)	2,415	5 (4, 6)	2,107	5 (4, 6)	1,864	4 (3, 6)	7,935	5 (4, 6)
**Place of delivery**	1,746		2,506		2,143		2,083		8,478	
Institutional		1,527 (87.5%)		2,412 (96.2%)		1,892 (88.3%)		1,988 (95.4%)		7,819 (92.2%)
Home		219 (12.5%)		94 (3.8%)		251 (11.7%)		95 (4.6%)		659 (7.8%)
**Consulted ANC (self report)**	1,782		2,513		2,160		2,064		8,519	
Yes		1,619 (90.9%)		2,466 (98.1%)		2,147 (99.4%)		1,960 (95.0%)		8,192 (96.2%)
No		163 (9.1%)		47 (1.9%)		13 (0.6%)		104 (5.0%)		327 (3.8%)
**HIV test during ANC** a	1,563		2,418		2,118		1,868		7,967	
Yes		607 (38.8%)		2,242 (92.7%)		1,924 (90.8%)		1,748 (93.6%)		6,521 (81.9%)
No		688 (44.0%)		148 (6.1%)		189 (8.9%)		103 (5.5%)		1,128 (14.2%)
Do not know		268 (15.5%)		28 (1.1%)		5 (0.2%)		17 (0.9%)		318 (3.8%)
**HIV test result (self report)** b	606		2,235		1,919		1,741		6,501	
HIV-negative		555 (91.6%)		2,088 (93.4%)		1,639 (85.4%)		1,531 (87.9%)		5,813 (89.4%)
HIV-positive		2 (0.3%)		110 (4.9%)		242 (12.6%)		158 (9.1%)		512 (7.9%)
Do not know		47 (7.8%)		35 (1.6%)		36 (1.9%)		39 (2.2%)		157 (2.4%)
Refused to answer		2 (0.3%)		2 (0.1%)		2 (0.1%)		13 (0.7%)		19 (0.3%)
**Birth weight (median [IQR])**	1,356	3,000 (2,700, 3,350)	2,073	3,300 (3,000, 3,600)	1,819	3,100 (2,800, 3,400)	1,914	3,020 (2,700, 3,360)	7,162	3,100 (2,800, 3,500)
>2,500 g		1,130 (83.3%)		1,863 (89.9%)		1,592 (87.5%)		1,611 (84.2%)		6,196 (86.5%)
≤2,500 g		226 (16.7%)		210 (10.1%)		227 (12.5%)		303 (15.8%)		966 (13.5%)
**Measles vaccination (children>10 mo only)**	741		1,086		1,165		1,143		4,135	
Yes		452 (61.0%)		975 (89.8%)		1,004 (86.2%)		931 (81.5%)		3,362 (81.3%)
No		289 (39.0%)		111 (10.2%)		161 (13.8%)		212 (18.5%)		773 (18.7%)
**Bacillus Calmette–Guerin vaccination**	1,412		2,391		2,144		2,049		7,996	
Yes		1,259 (89.2%)		2,286 (95.6%)		1,975 (92.1%)		2,000 (97.6%)		7,520 (94.0%)
No		153 (10.8%)		105 (4.4%)		169 (7.9%)		49 (2.4%)		476 (6.0%)
**Child feeding method during first 6 mo (limited to children >6 mo of age)**	1,250		1,579		1,429		1,515		5,773	
Exclusive breastfeeding		1,170 (93.6%)		1,112 (70.4%)		1,217 (85.2%)		589 (38.9%)		4,088 (70.8%)
Mixed		73 (5.8%)		449 (28.4%)		201 (14.1%)		713 (47.1%)		1,436 (24.9%)
Formula		7 (0.6%)		18 (1.1%)		11 (0.8%)		213 (14.1%)		249 (4.3%)

Data are number (percent) unless otherwise indicated.

a Restricted to those who consulted ANC.

b Restricted to those who consulted ANC and had an HIV test.

ANC, antenatal care.

The median birth weight of the index pregnancy was similar across countries (total cohort median 3,100 g; IQR: 2,800–3,500). Measles vaccination coverage by 10 mo of age ranged between 61% (Côte D'Ivoire) and 90% (Cameroon). In Côte D'Ivoire, Cameroon, and Zambia, more than 75% of children more than 6 mo of age were reported to have been exclusively breastfed for 6 mo, while in South Africa, only 40% of children were reported to have been exclusively breastfed, and mixed feeding was common (47%). The proportion of HIV-exposed infants in each country who received no PMTCT prophylaxis at all varied from 48% in Cameroon to 61% in South Africa. The highest proportion of infants whose mothers received HAART during pregnancy was found in Côte D'Ivoire (46%), followed by 14% in Zambia, 9% in South Africa, and 4% in Cameroon (Table 2).

### HIV-Free Survival

**Figure 2:**
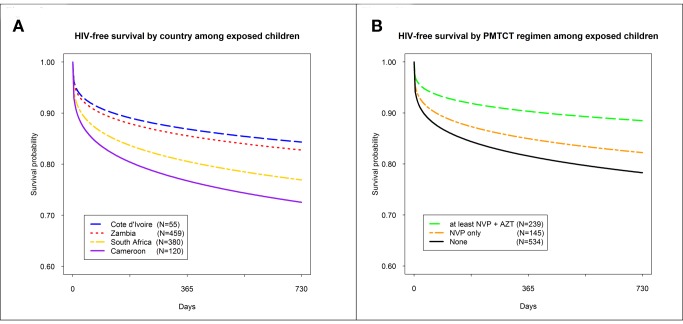
HIV-free survival by country and by PMTCT regimen in the PEARL Study. (A) HIV-free survival by country among exposes children; (B) HIV-free survival by PMTCT regimen among exposed children. AZT, zidovudine.

HIV-free survival at 24 mo of age among HIV-exposed children was 79.7% (95% CI: 76.4, 82.6). These rates differed by country, but not significantly so: Cameroon (72.6%; 95% CI: 62.3, 80.5), South Africa (77.7%; 95% CI: 72.5, 82.1), Zambia (83.1%; 95% CI: 78.4, 86.8), and Côte D'Ivoire (84.4%; 95% CI: 70.0, 92.2) (Figure 2A). HIV-free survival was highest in children born to mothers who received dual ARV prophylaxis or HAART (88.5%; 95% CI: 82.4, 92.6), followed by children whose mothers received single-dose nevirapine (NVP) (82.2%; 95% CI: 73.7, 88.2), followed by children who received no PMTCT intervention (78.3%; 95% CI: 73.9, 82.0), although these results did not differ significantly (Figure 2B). In adjusted analyses, risk of death or HIV infection among children receiving a more complex regimen of either dual ARV prophylaxis or HAART was lower (adjusted HR: 0.60; 95% CI: 0.34, 1.06; *p* = 0.067), compared to those receiving no prophylaxis, but not significantly so. In a pairwise comparison of children receiving a more complex regimen of either dual ARV prophylaxis or HAART versus those receiving only single-dose NVP prophylaxis, we did not observe additional benefit (adjusted HR: 0.88; 95% CI: 0.45, 1.44), but power to detect a difference was limited. No other parameters studied were associated with improved HIV-free survival (Table 4).

**Table 4:** Adjusted hazard of HIV infection or death among HIV-exposed children under 2 y of age in the PEARL Study (*n* = 893).

Category	Characteristic	Adjusted HR (95%CI)
**Household**	**Toilet facilities in household**	
	Flush toilet or pit latrine	1.0
	No facility	1.3 (0.8, 2.1)
	**Main material of floor**	
	Finished floor (cement/tiles/wood planks)	1.0
	Natural floor (earth/mud/dung/sand)	0.9 (0.5, 1.5)
	**Does your household have a cell phone?**	
	Yes	1.0
	No	1.2 (0.8, 1.8)
**Mother**	**Age at time of survey (years)**	0.98 (0.94, 1.03)
	**School level**	
	No schooling or primary	1.4 (0.9, 2.2)
	Secondary or higher	1.0
**Infant**	**PMTCT prophylactic regimen**	
	Zidovudine+NVP or HAART	0.60 (0.34, 1.06)
	NVP only^a^	0.68 (0.39, 1.19)
	None	1.0
	**Child feeding method during first 6 mo of life (restricted to children older than 6 mo; ** ***n*** ** = 570)**	
	Exclusive breastfeeding	1.0
	Mixed	0.51 (0.19, 1.36)
	Formula	0.42 (0.12, 1.44)

Adjusted analyses were restricted to children with responses for each of the covariates of interest; all models adjust for country effects.

a Pairwise comparison of NVP only versus zidovudine+NVP or HAART adjusted HR: 0.88 (95% CI: 0.45, 1.72).

### PMTCT Intervention Coverage

Of 976 HIV-exposed infants in the study for whom complete laboratory and survey data are available, 449 reportedly received at least some ARV drug prophylaxis during pregnancy or the postnatal period. This represents an unadjusted community PMTCT coverage of 46% (95% CI: 43, 49). Community PMTCT coverage differed significantly by country. It was highest in Cameroon, where 75 of 114 HIV-exposed infants met criteria for coverage (66%; 95% CI: 56, 74), followed by Zambia (219 of 444, 49%; 95% CI: 45, 54), then South Africa (152 of 365, 42%; 95% CI: 37, 47), and then Côte D'Ivoire (3 of 53, 5.7%; 95% CI: 1.2, 16).

**Figure 3:**
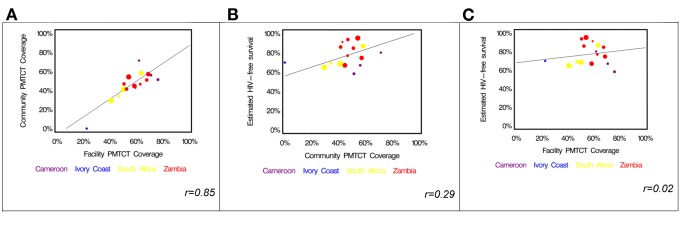
Relationship among service coverage at the facility level, service coverage at the community level, and HIV-free survival in children under 2 y of age in the PEARL Study. (A) Facility-based coverage versus community-based coverage; (B) community-based coverage versus HIV-free survival; (C) facility-based coverage versus HIV-free survival.

In a cluster-level analysis limited to the 16 facility–community pairs that had at least 15 HIV-exposed infants, we found community PMTCT coverage to be highly correlated with facility PMTCT coverage [15] (Pearson's correlation coefficient *r* = 0.85). In every instance except for two, the facility-based coverage measure exceeded that observed in the community (Table 5; Figure 3A). While community PMTCT coverage was moderately correlated with HIV-free survival (*r* = 0.29; Figure 3B), we did not observe an association between facility PMTCT coverage and community HIV-free survival (*r* = 0.02; Figure 3C).

**Table 5:** Comparison of PMTCT service coverage in the community survey compared to results of a simultaneous survey in corresponding facilities [15].

Country (Site)	Community		Facility	
	*n*/*N*	Percent Coverage (95% CI)	*n*/*N*	Percent Coverage (95% CI)
Cameroon (1)	23/41	56.1 (39.7–71.5)	186/262	71.0 (65.1–76.4)
Cameroon (2)	18/35	51.4 (34.0–68.6)	61/80	76.3 (65.4–85.1)
Cameroon (3)	20/28	71.4 (51.3–86.8)	135/218	61.9 (55.1–68.4)
Côte D'Ivoire (1)	0/18	0.0 (0.0–18.5)	26/116	22.4 (15.2–31.1)
South Africa (1)	51/124	41.1 (32.4–50.3)	89/177	50.3 (42.7–57.9)
South Africa (2)	53/91	58.2 (47.4–68.5)	124/195	63.6 (56.4–70.3)
South Africa (3)	12/35	34.3 (19.1–52.2)	51/109	46.8 (37.2–56.6)
South Africa (4)	34/116	29.3 (21.2–38.5)	76/186	40.9 (33.7–48.3)
Zambia (1)	14/30	46.7 (28.3–65.7)	22/35	62.9 (44.9–78.5)
Zambia (2)	57/105	54.3 (44.3–64.0)	72/133	54.1 (45.3–62.8)
Zambia (3)	6/14	42.9 (17.7–71.1)	32/54	59.3 (45.0–72.4)
Zambia (4)	22/53	41.5 (28.1–55.9)	53/101	52.5 (42.3–62.5)
Zambia (5)	25/49	51.0 (36.3–65.6)	42/62	67.7 (54.7–79.1)
Zambia (6)	38/85	44.7 (33.9–55.9)	83/142	58.5 (49.9–66.7)
Zambia (7)	44/77	57.1 (45.4–68.4)	115/167	68.9 (61.2–75.8)
Zambia (8)	23/49	46.9 (32.5–61.7)	83/164	50.6 (42.7–58.5)
Crude^a^ mean	440/950	46.3 (43.2–49.5)	1,250/2,201	56.8 (54.7–58.9)

Coverage defined as the proportion of children born to HIV-positive mothers in whom any ARV drug is reported to have been used during pregnancy.

a Not adjusted to account for clustering within communities or countries.

## Discussion

The PEARL Study community survey sampled representative households in four African countries to estimate 24-mo HIV-free survival among HIV-exposed children. Despite high reported HIV testing rates and utilization of delivery services, overall community PMTCT coverage was low, with less than half of HIV-exposed infants having received any ARV prophylaxis during gestation or the postnatal period. We think there is an important lesson to be learned in comparing the previously reported facility-based coverage estimates [15] to the community-based estimates reported here. While the estimates correlate highly (*r* = 0.85), we found the facility-based coverage estimate to be higher than the community-based coverage estimate in every facility–community pair except for two (Figure 3A). This suggests to us that the facility population can be thought of as a subset of the community, and a coverage estimate derived from it will be valid only in settings where most patients come to the facility for services. There are mother–infant pairs in the community who are simply not sampled by a facility-based estimate, and, not surprisingly, the PMTCT coverage rates among these women were generally lower. This finding is consistent with the observed associations between the two PMTCT coverage estimates and HIV-free survival (Figure 3B and 3C). While there is a moderate association between community PMTCT coverage and HIV-free survival, the association weakens considerably when the coverage estimate is derived from the facility population only.

The risk of death was lower among children using a potent ARV regime for PMTCT, but due to limited power, the association was not significant. An effect of drug potency is suggested by a dose response in the adjusted analysis and in Figure 2B. While this phenomenon has been known for some time in the controlled environment of clinical trials, its demonstration at the population level provides support for World Health Organization recommendations to implement more complex PMTCT ARV regimens on a wide scale.

In aggregate, we think our findings suggest a critical role for both facility- and community-based monitoring of PMTCT programs. The facility-based estimates—particularly those capable of recreating the PMTCT “cascade” [15],[17]—can provide important, practical information about how to best improve service delivery at the site level. Facility-based estimates are also easier to obtain, and are thus suited for more routine performance monitoring. Since some patients do not interact with the healthcare system at the level of the facility, however, periodic evaluation of community PMTCT coverage must also be considered. This aspect of monitoring is most useful when utilization of facility-based services is low, but is also subject to bias if sampling is not random. In our study, for instance, we used random sampling, but excluded those sites and communities known not to have any PMTCT services at all. Thus, both our facility and community samples are optimistic representations.

We observed an overall rate of HIV-free survival that was higher than anticipated by our original modeling [6]. This could be explained by at least three sources of bias. First, all infant deaths in the communities may not have been identified in the survey. While every attempt was made to ensure complete ascertainment, including use of census enumerators from the local community and study instruments that queried child deaths in multiple ways, we cannot be certain that all deaths were captured. Second, because we chose to sample only those communities that had at least some access to PMTCT services, the included clusters may have not been representative of the population as a whole. Since better-resourced facilities will be the first to implement PMTCT services, our sampled patients may have had access to better services than others in their respective countries. This suspicion is supported by the finding that over 85% of respondents reported having delivered at a facility, which is higher than we expected from national figures [23]–[26] but which is perhaps explained by our selection of households based on their proximity to particular health facilities. A third potential bias of our study lies in its imperfect participant response rates. Although a response rate of 78% is typical for a community-based survey, households where occupants were not found or refused to participate may have been systematically different from those that were included. If a household with a death under 24 mo of age was more likely to refuse entry to study staff, this could have resulted in overestimation of HIV-free survival, especially if that refusal was more likely among households affected by HIV.

The questionnaire instruments and sampling methodology for this study drew heavily on the widely used DHS surveys [22]. The successful conduct of the PEARL Study, and others of similar design [27], demonstrates that with relatively minor modification, the DHS surveys could serve as a platform for widespread estimation of PMTCT program effectiveness. The most critical modification would require testing mothers for HIV antibodies (to ascertain infant HIV exposure) and, for those who are seropositive, testing their infants for infection. Some countries already perform HIV testing as part of their DHS surveys, but others do not, and this modification would have to be planned and resources for its implementation identified.

Monitoring PMTCT programs remains a formidable challenge. Given the continually increasing emphasis on program scale-up, the unprecedented donor investment in affected countries, and the recent call by the World Health Organization and others for the virtual elimination of HIV in children by 2015, it is imperative that the international research and policy communities agree upon standard methodologies to measure the effectiveness of programs over time. Population-based evaluations of PMTCT effectiveness placed within periodic national health surveys could represent an important program monitoring resource.

## Abbreviations

ARV: antiretroviral
DHS: Demographic and Health Surveys
HAART: highly active antiretroviral therapy
HR: hazard ratio
IQR: interquartile range
NVP: nevirapine
PEARL Study: PMTCT Effectiveness in Africa: Research and Linkages to Care Study
PMTCT: prevention of mother-to-child HIV transmission
